# Identification of Tumor Antigens and Immune Subtypes for the Development of mRNA Vaccines and Individualized Immunotherapy in Soft Tissue Sarcoma

**DOI:** 10.3390/cancers14020448

**Published:** 2022-01-17

**Authors:** Changwu Wu, Yingjuan Duan, Siming Gong, Georg Osterhoff, Sonja Kallendrusch, Nikolas Schopow

**Affiliations:** 1 Institute of Anatomy, University of Leipzig, 04103 Leipzig, Germany; changwu.wu@studserv.uni-leipzig.de (C.W.); sonja.kallendrusch@medizin.uni-leipzig.de (S.K.); nikolas.schopow@medizin.uni-leipzig.de (N.S.); 2 Faculty of Chemistry and Mineralogy, University of Leipzig, 04103 Leipzig, Germany; yingjuan.duan@studserv.uni-leipzig.de; 3 Sarcoma Center, Department of Orthopedics, Trauma and Plastic Surgery, University Hospital Leipzig, 04103 Leipzig, Germany; georg.osterhoff@medizin.uni-leipzig.de; 4 Faculty of Medicine, Health and Medical University Potsdam, 14471 Potsdam, Germany

**Keywords:** mRNA vaccine, soft tissue sarcoma, immune subtypes, individualized immunotherapy, tumor immune microenvironment

## Abstract

**Simple Summary:**

Soft tissue sarcomas (STS) are a group of rare malignant tumors with high tissue heterogeneity and poor prognosis, and which are still without effective individualized immunotherapy approaches. In this study, four potential tumor antigens, six STS immune subtypes, and six functional gene modules were identified. The different immune subtypes have different molecular, cellular, and clinical characteristics. The superiority of mRNA vaccine therapies has been demonstrated during the current pandemic as well as in tumor vaccine studies, and the present study provides guidance for future mRNA vaccine development. Furthermore, in future individualized immunotherapies for STS, it is possible to select different immunotherapies based on the different immune subtypes identified in this study. In fact, the immune subtypes identified in this study explain, to some extent, the failure of immunotherapy for certain STS subtypes in previous clinical trials, and facilitate further understanding of strategy selection for the immunotherapy of STS. To our knowledge, this is the first study to address STS mRNA vaccine development and immunophenotyping. This study provides a theoretical framework for STS mRNA vaccine development and the selection of patients for vaccination and provides a reference for promoting individualized immunotherapy.

**Abstract:**

Soft tissue sarcomas (STS) are a rare disease with high recurrence rates and poor prognosis. Missing therapy options together with the high heterogeneity of this tumor type gives impetus to the development of individualized treatment approaches. This study identifies potential tumor antigens for the development of mRNA tumor vaccines for STS and explores potential immune subtypes, stratifying patients for immunotherapy. RNA-sequencing data and clinical information were extracted from 189 STS samples from The Cancer Genome Atlas (TCGA) and microarray data were extracted from 103 STS samples from the Gene Expression Omnibus (GEO). Potential tumor antigens were identified using cBioportal, the Oncomine database, and prognostic analyses. Consensus clustering was used to define immune subtypes and immune gene modules, and graph learning-based dimensionality reduction analysis was used to depict the immune landscape. Finally, four potential tumor antigens were identified, each related to prognosis and antigen-presenting cell infiltration in STS: HLTF, ITGA10, PLCG1, and TTC3. Six immune subtypes and six gene modules were defined and validated in an independent cohort. The different immune subtypes have different molecular, cellular, and clinical characteristics. The immune landscape of STS reveals the immunity-related distribution of patients and intra-cluster heterogeneity of immune subtypes. This study provides a theoretical framework for STS mRNA vaccine development and the selection of patients for vaccination, and provides a reference for promoting individualized immunotherapy.

## 1. Introduction

Soft tissue sarcomas (STS) are a highly heterogeneous group of malignant tumors with more than 100 identified subtypes [1]. The most common types are liposarcoma, including dedifferentiated liposarcoma (DDLPS), leiomyosarcoma (LMS), myxofibrosarcoma (MFS), undifferentiated pleomorphic sarcoma (UPS), and synovial sarcoma (SS) [2]. All STS subtypes represent only about 1% of all adult solid tumors [3]. For patients with localized tumors, surgery is the primary treatment, and postoperative radiotherapy is beneficial for improving the local control rate, while cytotoxic chemotherapy is often used for patients with recurrences or metastases [4,5,6]. The 5-year survival rates range from 67.1–73.7% for STS of limbs to 38.2–45.0% for STS of peritoneum and retroperitoneum in Europe and North America [7]. In a recent meta-analysis, with a total of 3157 patients, adjuvant chemotherapy showed neither improvement in OS nor progressive free survival either in all STS or in subgroups [8]. New treatment strategies are urgently needed to improve the prognosis of patients with STS.

Immunotherapy, especially immune checkpoint inhibitor (ICI) therapy, has achieved considerable benefits in diverse solid tumors, such as melanoma [9,10,11,12,13]. However, not all patients benefit from ICI [12]. For example, in a multicenter trial of pembrolizumab (PD-1 inhibitor) for STS, 40% of patients with UPS and 20% of patients with DDLPS showed appreciable responses, while only one in ten patients with SS responded, and all patients with LMS were non-responders [14].

Another immunotherapeutic strategy that has attracted significant attention is the use of tumor vaccines, which have many advantages, such as being more specific, inducing durable immunity, and effectively overcoming the limited therapeutic efficacy, drug resistance, and adverse effects associated with conventional chemotherapy or ICI [15,16]. Tumor vaccines are mainly divided into therapeutic and preventive vaccines. Therapeutic tumor vaccines are often used in the advanced cancer to remove cancer cells, while preventive tumor vaccines can be used in healthy people to prevent cancerogenic infections (e.g., human papillomavirus or hepatitis B virus) [17]. In this study, we focused on therapeutic vaccines, which promote a specific anti-tumor immune response [17,18]. There are currently four main types of tumor vaccines: tumor or immune cell vaccines, peptide vaccines, viral vector vaccines, and DNA or RNA vaccines [18]. Early studies of STS tumor vaccines focused on tumor cells as antigens. In one study, the researchers vaccinated STS patients with irradiated autologous tumor cells along with the adjuvants GM-CSF or IFN-γ. Although there was no objective tumor response, the median OS of the patients was significantly prolonged for 8.6 months [19]. In subsequent studies, the feasibility of the SYT-SSX-derived peptide vaccine in SS was confirmed, but no objective response was observed [20,21]. Compared with several other types of vaccines, messenger RNA (mRNA) vaccines have obvious advantages [22]. First, unlike peptide vaccines, mRNA vaccines are less restricted by the types of human leukocyte antigens, which can stimulate a wider range of T cell responses [23]. Second, mRNA vaccines can deliver multiple tumor-associated antigens (TAAs) or tumor-specific antigens (TSAs) simultaneously and induce both cellular and humoral immune responses [22]. Compared to DNA vaccines, mRNA vaccines do not have the risk of insertional mutations by integration into the genome. Furthermore, mRNA vaccines have a good safety profile and an efficient production rate [22,24,25]. mRNA sequences are easy to modify to encode with any antigen, and in this way the mRNA vaccine production line of a certain antigen can be quickly and easily transformed into the production line of another antigen, which is very important for personalized treatment [22,26]. The huge advantages of mRNA vaccines in the COVID-19 pandemic further confirm their superiority, and this has aroused widespread interest in the field of tumor treatment and infectious diseases [27,28,29].

So far, mRNA tumor vaccines have achieved promising results in non-small-cell lung cancer, prostate cancer, colorectal cancer, and melanoma, among others [30,31,32,33,34]. However, mRNA vaccines against STS have not yet been developed. Given the high level of tumor heterogeneity and the complex tumor immune microenvironment (TIME) in STS, patients to be treated should be carefully selected in advance.

The purpose of this study is to explore tumor antigens for the development of STS mRNA vaccines, and to identify potential immune subtypes (ISs) and immune landscape in STS to determine the population suitable for different types of immunotherapy, including mRNA vaccines.

## 2. Materials and Methods

### 2.1. Patients

The RNA-sequencing data and clinical information of 189 STS samples were obtained from TCGA database through the Xena Functional Genomics Explorer (University of California, Santa Cruz, https://xenabrowser.net/datapages/ (accessed on 5 May 2021)) and a publication of TCGA Research Network was used as a discovery cohort [35], which included DDLPS, LMS, MFS, MPNST, SS, and UPS (Table S1). The microarray data of 103 STS samples in the GSE21122 dataset were obtained through the GEO database (https://www.ncbi.nlm.nih.gov/geo/ (accessed on 5 May 2021)) as an independent validation cohort [36]. Both RNA-seq data and microarray data were normalized using the log2(x + 1) transformation, and samples without complete clinical information, as well as genes with low expression abundance, were excluded. In addition, the VarScan-processed mutation dataset in TCGA cohort was obtained from Genomic Data Commons Data Portal (https://portal.gdc.cancer.gov/ (accessed on 10 May 2021)).

### 2.2. Identification of Potential Tumor Antigens

To identify and visualize genetic variants in STS, and to screen for amplified and mutated genes, the cBioPortal (https://www.cbioportal.org/ (accessed on 10 May 2021)) tool was used. In addition, the genetic alterations of STS in TCGA cohort were summarized and visualized using the R package “maftools” in the R software (Version 4.0.3, R Foundation for Statistical Computing, Vienna, Austria). To recognize potential tumor antigens closely related to the OS of STS, the R package “rbsurv” was used for robust likelihood-based survival analysis on amplified and mutated genes. Patients were divided into low- and high-expression groups using the median expression of candidate genes as cut-off values, and the relationship between candidate genes and OS and relapse free survival (RFS) in STS patients was visualized and evaluated using the Kaplan–Meier curve and the Logrank test. In addition, the Oncomine database (https://oncomine.org/ (accessed on 25 May 2021)) was used to identify candidate genes that were overexpressed in tumor tissues compared to normal tissues.

To explore the correlation between candidate tumor antigens and the infiltration of antigen-presenting cells (APCs), this study used the single sample gene set enrichment analysis (ssGSEA) to estimate the infiltration levels of dendritic cells and macrophages in individual STS samples [37], followed by the Pearson correlation test to determine the R and *p*-values of correlation between candidate genes expression and infiltration of APCs. Statistical significance was set at *p* < 0.05 in all statistical analyses.

### 2.3. Identification and Validation of Immune Subtypes

First, 662 immune-related genes expressed in TCGA cohort were identified from the ImmPort database [38], of which 606 were also expressed in the GEO cohort. Subsequently, based on the expression of immune-related genes, the partition around medoids (PAM) algorithm was used to perform unsupervised consensus clustering to identify potential STS patient clusters in the discovering cohort. A total of 1000 bootstraps were performed, and each bootstrap included 80% of the patients, with the number of clusters set at 2–10. The optimal number of clusters was defined by evaluating the consensus cumulative distribution function, delta area, and consensus heatmap [39]. The same settings were used in the validation cohort to identify potential ISs to determine the repeatability of the ISs. In addition, intra-group proportion (IGP) analysis was performed to assess the reproducibility and similarity of the ISs between the discovery and validation cohorts [40].

### 2.4. Molecular and Cellular Characteristics Related to Immune Subtypes

In this study, ssGSEA was performed using the R package “GSVR” to quantify the enrichment score of the 28 previously reported immune cells in each sample [36]. In addition, the relationship between the ISs and 56 previously reported immune-related molecular and cellular characteristics was assessed [41]. Student’s *t* test or an ANOVA was used to determine significance.

### 2.5. Defining the Immune Landscape

Considering the heterogeneity of the STS and the dynamic nature of the immune system, this study used the reduceDimension function in the R package “Monocle” to perform a discriminative dimensionality reduction of trees (DDRTree), with the maximum component set to four, and the plot cell trajectory function was used for visualization [42]. This method is a graph learning-based dimensionality reduction method that reflects the relationship among patients in a nonlinear manifold and complements the previously defined ISs.

### 2.6. Identification of Immune-Related Gene Modules and Hub Genes

Based on the immune-related gene expression profile, consensus clustering was performed using the same settings and parameters as described above for identifying potential ISs to identify robust immune gene modules (GMs). Next, the biological functions of each GM were annotated according to gene ontology (GO) biological processes by the R package “clusterProfiler”. The GM score was defined as the average expression level of all genes in a specific GM. GMs in the validation cohort were defined using the same gene arrangement as in the discovery cohort. Using age, Fédération Nationale des Centres de Lutte Contre le Cancer (FNCLCC) grade, gender, radiotherapy, pharmaceutical therapy, and pathological tumor size as covariates, multivariate cox regression analyses were performed to evaluate the relationship between GM score and OS and RFS. In addition, the STRING tool (https://string-db.org/ (accessed on 25 May 2021)) was used to construct a protein–protein interaction (PPI) network for the genes in GM3 [43]. Then, the top 10 hub genes in the PPI network were identified using the cytoHubba plugin in the Cytoscape software (version 3.8.2, https://cytoscape.org/index.html, accessed on 13 May 2021) [44].

## 3. Results

### 3.1. Identification of Potential Tumor Antigens in STS

**Figure 1:**
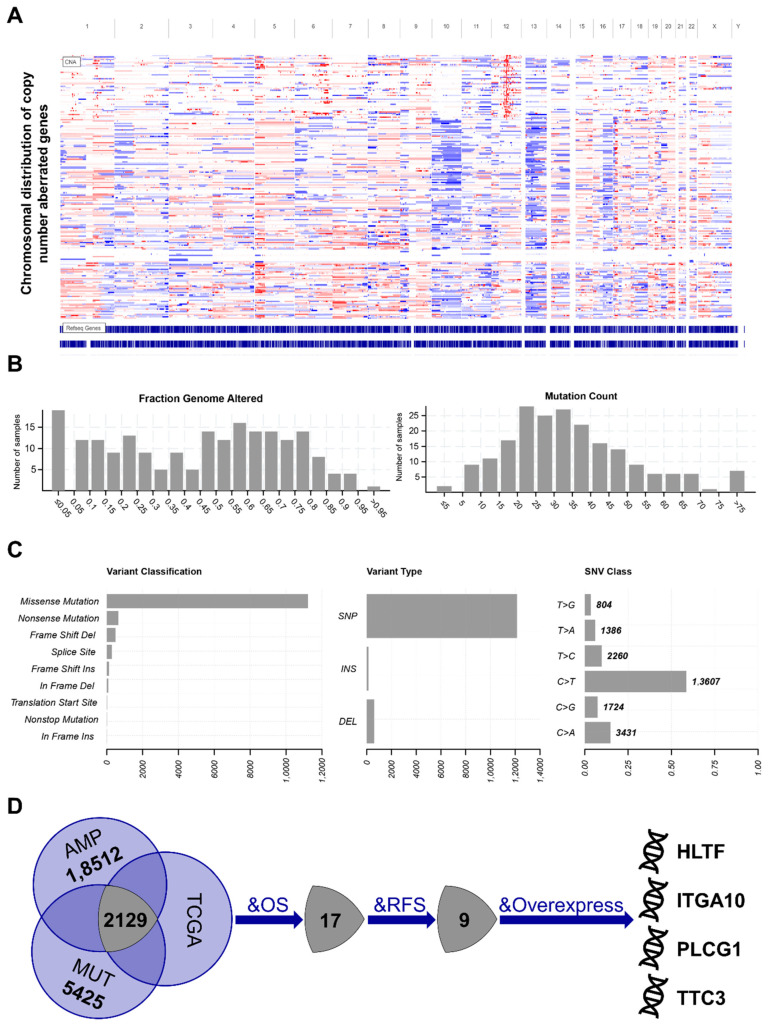
Identification of potential tumor antigens in STS. (**A**) The chromosomal distribution of the gene copy number aberrations in STS. (**B**) Samples in altered genome fraction and mutation count groups. (**C**) Summary of the mutation information for STS. (**D**) The intersection of amplified (AMP: 18,512) and mutated (MUT: 5425) genes, of which 2129 were detected in TCGA. In total, four of these potential tumor antigens were significantly associated with OS and RFS prognosis and were overexpressed.

The setup of this study is shown in Figure S1. To identify potential tumor antigens in STS, the genes with copy number variation in STS were screened and 18,512 amplified genes were obtained (Figure 1A). Since the amplification of genes usually results in gene overexpression, some of these genes may be able to encode TAAs, which requires further narrow-down analysis. By evaluating the genetic mutations in STS patients, 5425 mutated genes that may encode TSAs were then screened. Finally, 4622 amplified and mutated genes were screened out, and 2129 of them were detected in TCGA discovering cohort. It was found that most STS patients had moderate fractional genomic changes and mutation counts (Figure 1B), indicating that STS has medium immunogenicity. In addition, it was found that missense mutations accounted for the largest proportion of all mutation categories, compared with insertions and deletions, and that single nucleotide polymorphisms occurred most frequently, while C > T is the most common single nucleotide variation in STS (Figure 1C).

**Figure 2:**
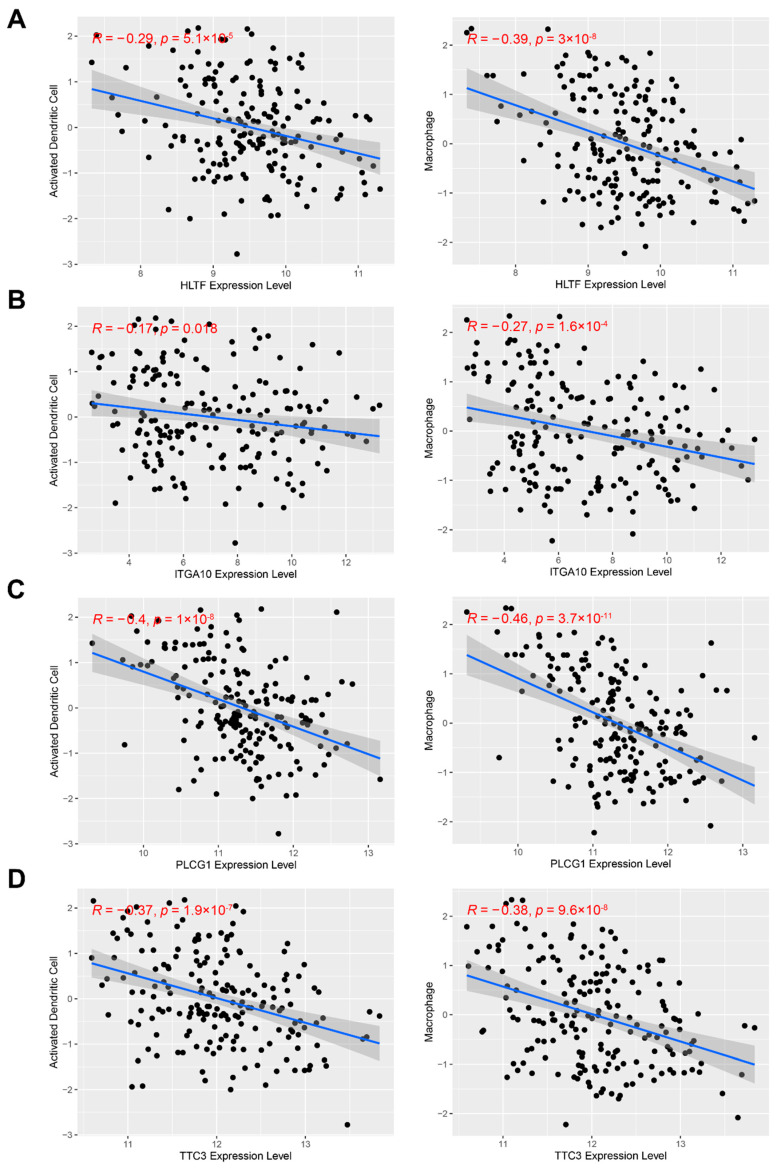
Identification of tumor antigens associated with the infiltration of antigen-presenting cells in TCGA cohort. (**A**–**D**) Correlation between HLTF (**A**), ITGA10 (**B**), PLCG1 (**C**), and TTC3 (**D**) expression and infiltration levels of dendritic cells and macrophages in STS.

To identify potential antigens that play a key role in STS, the prognostic value of the above-mentioned genes was explored. From these, 17 genes were identified that were strongly associated with the OS of STS patients, and nine of which were significantly associated with RFS (Figure 1D). To further determine the importance of these nine genes in STS, their expression levels in STS tissues were explored through the Oncomine database, and four of them were significantly overexpressed compared to normal tissues: helicase-like transcription factor (HLTF), integrin subunit alpha 10 (ITGA10), phospholipase C gamma 1 (PLCG1), and tetratricopeptide repeat domain 3 (TTC3) (Figure S2). Patients with higher expressions of HLTF, ITGA10, PLCG1, and TTC3 had significantly worse OS and RFS (Figure S3). Notably, the expression of each of the four genes in STS was associated with the infiltration of dendritic cells and macrophages (Figure 2A–D). Although the correlation coefficients were only in the range of −0.17 to −0.46 due to the interference of other factors, it still suggested that these four tumor antigens may be directly processed and presented by APCs. This result was also verified in the GEO cohort (Figure S4). Overall, four genes were identified that are amplified, mutated, overexpressed, and strongly associated with STS prognosis as well as the infiltration of APCs. They are promising candidates for the development of mRNA vaccines against STS.

### 3.2. Identification of Potential Immune Subtypes in STS

**Figure 3:**
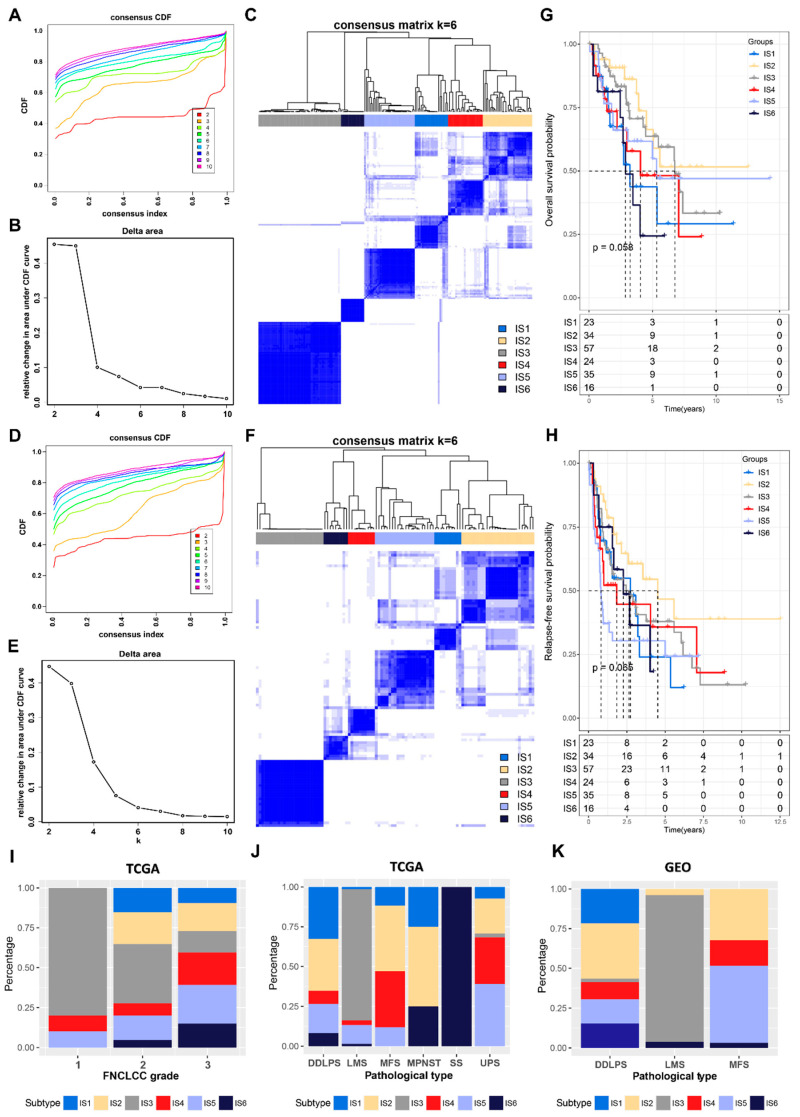
Identification of potential immune subtypes in STS. (**A**–**F**) Cumulative distribution function curve (**A**,**D**), delta area curve (**B**,**E**), and consensus heatmap (**C**,**F**) based on immune-related gene expression profile in TCGA (**A**–**C**) and GEO (**D**–**F**) cohorts. (**G**,**H**) Kaplan–Meier curves showing overall survival (G) and relapse-free survival (H) of immune subtypes. The significance was calculated using a log-rank test. The grouping of STS samples is shown at the bottom of the charts. (**I**) Distribution of immune subtypes in STS patients with different FNCLCC grades in TCGA cohort. (**J**,**K**) Distribution of immune subtypes in STS patients with different pathological subtypes in TCGA (**J**) and GEO (**K**) cohorts.

Immunophenotyping can reflect the immune status of STS patients, which is conducive to the selection of mRNA vaccine-sensitive patients. A consensus clustering of 189 samples from TCGA cohort was performed based on an immune-related gene expression profile, and six robust ISs (IS1–IS6) were finally identified by analyzing the cumulative distribution function, delta area, and consensus heatmap (Figure 3A–C). Using the same settings for validation in an independent GEO cohort, six robust ISs were also identified (Figure 3D–F). Subsequently, the IGP analysis further determined the consistency and reproducibility of the ISs in both cohorts (Table S2). In addition, ISs were observed to be associated with different prognoses, with IS2 having the best OS and RFS (Figure 3G,H). The distribution of ISs in different FNCLCC grades showed that IS3 was predominant in patients with FNCLCC grade 1. The category of FNCLCC grade 2 and 3 contained all IS groups (Figure 3I). The distribution of the ISs among the different pathological subtypes of STS in TCGA cohort (Figure 3J) and the GEO cohort (Figure 3K) were similar: all SS displayed only IS6; in LMS, IS3 is dominating; and in DDLPS and MFS, multiple ISs were distributed.

### 3.3. The Relationship between Immune Subtypes and Mutation Status in STS

**Figure 4:**
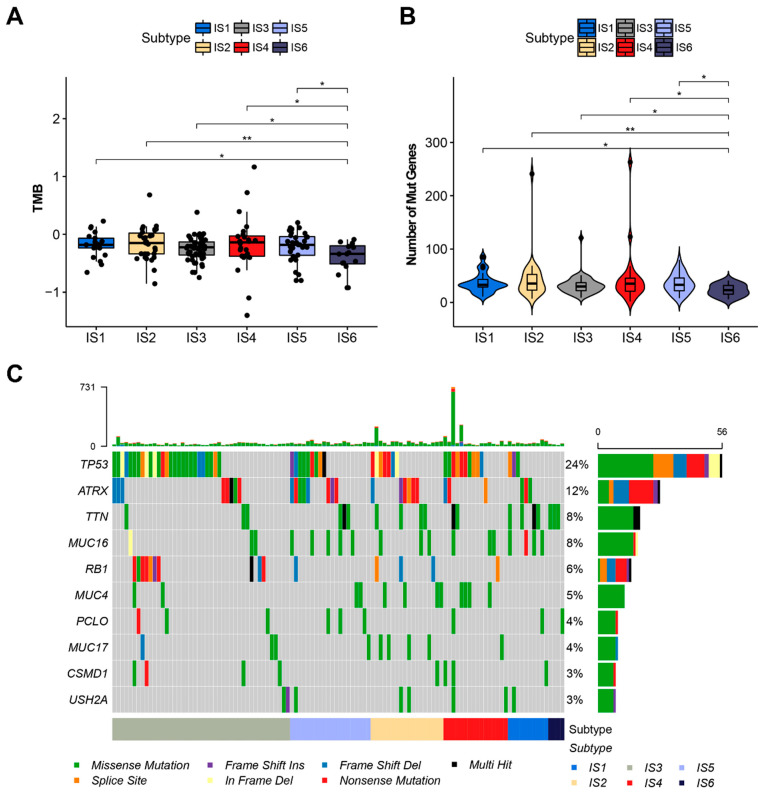
Association of immune subtypes with TMB and mutation in STS. (**A**,**B**) TMB (**A**) and mutation number (**B**) of different immune subtypes in STS. (**C**) The top 10 frequently mutated genes in STS immune subtypes. * *p* < 0.05 and ** *p* < 0.01.

The tumor mutation burden (TMB), as well as the number of mutations in tumor patients, are closely related to the effectiveness of tumor immunotherapy [45]. Here, TMB and the number of mutations in individual patients in different ISs were analyzed using the VarScan-processed mutation dataset derived from TCGA cohort. As shown in Figure 4A, IS6 patients had a significantly lower TMB compared to patients with other immune subtypes, and there are no statistical differences between IS1 and IS5. Similar results were also observed in the analyses of the number of mutated genes (Figure 4B). In addition, the top 10 most frequently mutated genes in the various ISs of STS, including TP53, are shown in Figure 4C. These results indicate that IS6 may have a lower response to immunotherapy, such as ICI therapy.

### 3.4. The Relationship between Immune Subtypes and Immunomodulators in STS

Immune checkpoints (ICPs) and immunogenic cell death (ICD) modulators are known to be crucial in tumor immunity and have a great impact on immunotherapy [46]. As shown in Figure S5A, 40 ICP-related genes were detected in TCGA cohort, of which 39 had a differential expression between ISs. For example, most ICP-related genes, including CTLA4, were significantly elevated in IS2 and IS4 tumors, in contrast to the significantly lower expression in IS6 tumors. In the GEO cohort, 36 ICP-related genes were detected, 30 of which were expressed differentially in different ISs (Figure S5B). CD160, CD200, CD48, CD86, LAIR1, LGALS9, TNFRSF14, TNFRSF25, and TNFSF14 were elevated in IS2 and IS4 tumors, while CD40, CD80, CTLA4, ICOS, ICOSLG, NRP1, TNFRSF4, TNFRSF8, and TNFSF9 were significantly downregulated in IS3 tumors. In addition, 20 ICD-related genes were detected in TCGA cohort, 19 of which were all expressed differentially between ISs (Figure S5C), while 22 expressed ICD genes that were detected in the GEO cohort, 17 of which showed differential expression between ISs (Figure S5D). Among them, EIF2A, EIF2AK1, and HMGB1 were highly expressed in IS6 of TCGA cohort, whereas EIF2AK1, HGF, LRP1, and MET were highly expressed in IS6 of the GEO cohort. Thus, immunophenotyping reflects the expression levels of different ICPs and ICD modulators; therefore, it appears that ISs may be a potential tumor vaccine predictor. IS2 and IS4, which were highly expressed ICP-related genes, may not be suitable for mRNA vaccines due to their immunosuppressive properties.

### 3.5. Molecular and Cellular Characteristics of Immune Subtypes

**Figure 5:**
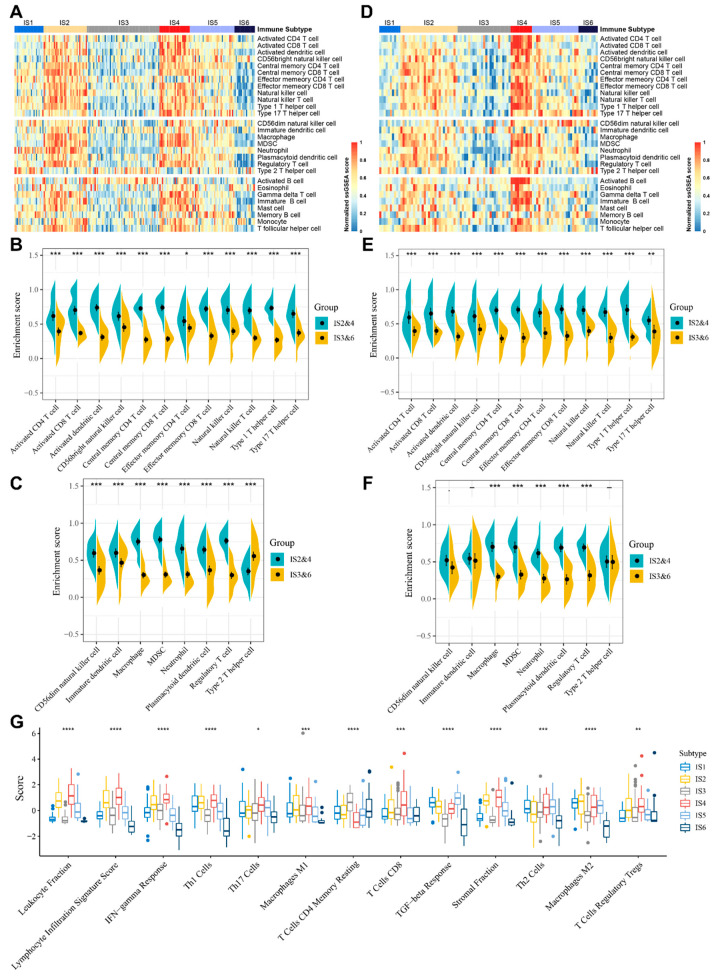
Cellular and molecular characteristic of immune subtypes in STS. (**A**,**D**) The heatmap of 28 previously reported immune cell signatures scores among STS immune subtypes in TCGA (**A**) and GEO (**D**) cohorts. (**B**,**C**) Differences for immunostimulatory cell (**B**) and immunosuppressive cell (**C**) signatures scores between IS2 and IS4 and IS3 and IS6 in TCGA cohort. (**E**,**F**) Differences for immunostimulatory cell (**E**) and immunosuppressive cell (**F**) signatures scores between IS2 and IS4 and IS3 and IS6 in GEO cohort. (**G**) Immune-related molecular signatures with significant differences among STS immune subtypes. − *p* ≥ 0.1, · *p* < 0.1, * *p* < 0.05, ** *p* < 0.01, *** *p* < 0.001, **** *p* < 0.0001.

The effect of mRNA vaccines in tumor patients is closely linked to the infiltration of immune cells in the TIME; therefore, the enrichment scores of 28 previously reported immune cells in STS were evaluated based on the ssGSEA method [37]. As shown in Figure 5A, in TCGA cohort, immune cells were clearly divided into six clusters based on the distribution of ISs. Interestingly, IS2 and IS4 had similar scores on immune cell infiltration, while IS3 had a similar distribution to IS6. In addition, IS2 and IS4 scored higher compared to IS3 and IS6 on all anti-tumor immunostimulatory cells, such as activated CD4+ T cells, activated CD8+ T cells, and natural killer cells (Figure 5B), as well as pro-tumor immunosuppressive cells, such as regulatory T (Treg) cells and myeloid-derived suppressor cells (MDSCs) (Figure 5C). Therefore, IS2 and IS4 are immunologically hot and immunosuppressive phenotypes, whereas IS3 and IS6 are immunologically cold phenotypes. IS1 and IS5 have relatively complex TIMEs (Figure 5A). Importantly, similar trends were also observed in the GEO cohort (Figure 5D–F).

In a landmark study, Thorsson et al. defined six pan-cancer immunotypes (C1-C6) by integrating more than 10,000 samples from 33 tumor types [40], of which STS included mainly C1 (wound healing), C2 (IFN-γ dominant), C3 (inflammatory), C4 (lymphocyte depleted) and C6 (TGF-β dominant). By comparing the distribution of this immunophenotype across our six ISs, it was found that C1 was predominant in IS5 and IS6, whereas C2 was predominant in IS4 (Figure S6A). C2 was dominated by IFN-γ and characterized by lymphocyte and CD8+ T cell infiltration, which is consistent with the previously described immune activation characteristics in IS4. In contrast, in other ISs, the pan-cancer immunophenotyping was more evenly distributed (Figure S6A). This demonstrates the difference in TIME between STS and the other 32 types of cancer.

The relationship between ISs and the 56 immune-related molecular signatures previously defined was evaluated. Here, 27 of these immune-related molecular signatures differed significantly between the ISs (Figure 5G and Figure S6B). Notably, IS2 and IS4 had the highest scores relating to leukocyte fraction, lymphocyte infiltration, IFN-γ response, Th1 cells, CD8+ T cells, stromal fraction, and Treg cells. Thus, IS2 and IS4 have an overall hot and suppressed TIME. In contrast, IS3 and IS6 had the lowest scores for leukocyte fraction, Th1 cells, stromal fraction, TGF-β response, and macrophage M2, with IS6 having the lowest scores for lymphocyte infiltration, IFN-γ response, Th17 cells, and Treg cells, reflecting the overall cold immune phenotype of IS3 and IS6. IS1 was characterized by a low score for leukocyte fraction, lymphocyte infiltration, and CD8+ T cells, and a high score for TGF-β response and macrophage M2, suggesting a colder and immunosuppressed immune state in IS1. IS5 exhibited an overall moderate immune cell infiltration, as well as a high score for TGF-β response, Th2 cells, and intratumor heterogeneity, indicating the presence of an immunosuppressive phenotype, as well as a complex TIME. Sarcoma patients who may benefit most from mRNA vaccination have cold immunophenotypes (IS3 and IS6), and an effective immune infiltration and immune response might be triggered here.

### 3.6. The Immune Landscape of STS

**Figure 6:**
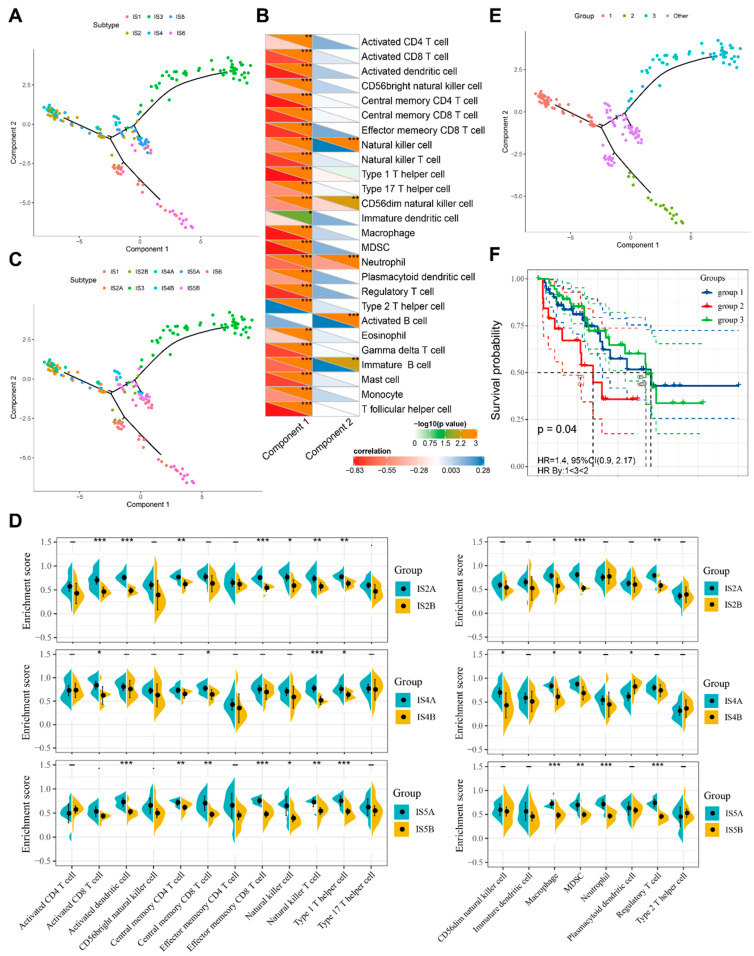
The immune landscape of STS. (**A**) Immune landscape of STS immune subtypes. Each dot represents a patient, and different colors represent different immune subtypes. The horizontal axis represents the principal component 1, and the vertical axis represents the principal component 2. (**B**) Correlation between principal components 1 and 2 and immune cell enrichment scores. (**C**) Immune landscape of the subgroups of STS immune subtypes. (**D**) Differences for immunostimulatory cell and immunosuppressive cell enrichment scores in the subgroups of STS immune subtypes. (**E**,**F**) Immune landscape of samples from three extreme locations (**E**) and their prognostic status (**F**). − *p* ≥ 0.1, · *p* < 0.1, * *p* < 0.05, ** *p* < 0.01, *** *p* < 0.001.

To reveal the immune distribution and immune components of individual patients based on the immune gene expression profile of STS patients, a graph learning-based dimensionality reduction method was used to define the immune landscape of STS (Figure 6A). STS patients in the immune landscape were divided into different clusters. The horizontal axis of the immune landscape displays the infiltration level of various anti-tumor immunostimulatory cells (activated CD8+ T cells, activated dendritic cells, natural killer cells, etc.) and immunosuppressive cells (MDSCs, Treg cells, etc.), while the vertical axis was correlated with the infiltration of natural killer cells, activated B cells, and immature B cells (Figure 6B). Consistent with this, the immunologically hot phenotypes, IS2 and IS4, were distributed opposite to the immune cold phenotypes, IS3 and IS6, in the immune landscape. In addition, the immune landscape also reflected the intra-cluster heterogeneity of ISs, according to the distribution of individual patients within the immune landscape. IS2, IS4, and IS5 were further divided into different subgroups (Figure 6C). The different subgroups had different immune cell infiltration levels. For example, IS2B had significantly lower enrichment scores for multiple immunostimulatory cells, including activated CD8+ T cells, activated dendritic cells, central memory CD4+ T cells, effector memory CD8+ T cells, and immunosuppressive cells, including MDSCs and Treg cells, than IS2A (Figure 6D). Similarly, IS4B has significantly lower enrichment scores for activated CD8+ T cells, central memory CD8+ T cells, natural killer T cells, Th17 cells, MDSCs, etc. IS5B scored lower in terms of activated dendritic cells, central memory CD4+ T cells, central memory CD8+ T cells, natural killer T cells, MDSCs, Th1 cells, Treg cells, etc. After further prognosis analysis of the patients located at extreme positions on the immune landscape, it was found that the prognosis is obviously different between different groups, and that group 2 has the poorest prognosis, which implies the prognostic predictive ability of the immune landscape (Figure 6E,F).

### 3.7. Identification of Functional Immune Gene Modules and Hub Genes in STS

**Figure 7:**
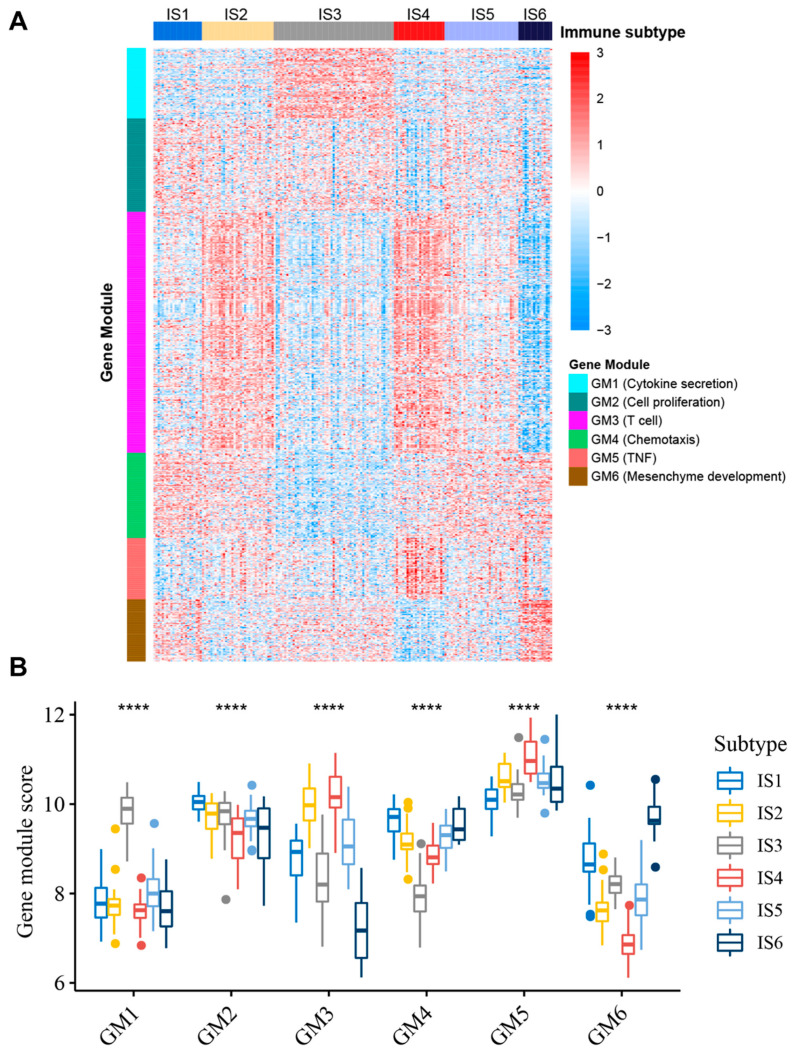
Functional immune genes modules. (**A**) Heatmap of ISs and GMs in STS patients in TCGA cohort. Genes are ordered based on the GM, and patients are arranged based on their ISs. (**B**) Box plots of the six GM expression patterns and ISs in TCGA cohort. **** *p* < 0.0001.

**Figure 8:**
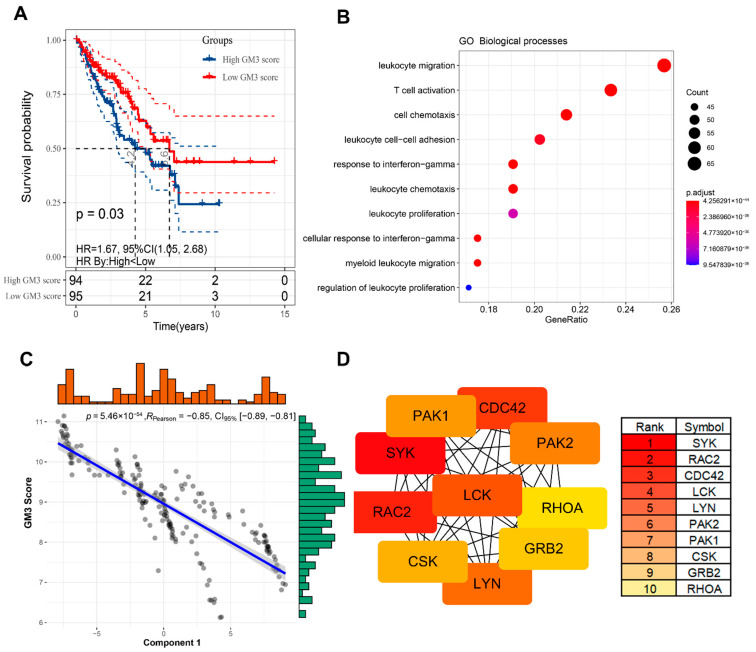
GM3 characteristics and immune hub genes. (**A**) Kaplan–Meier curve showing OS analysis of GM3 in STS. (**B**) GO biological process enrichment analysis of GM3. (**C**) Correlation between GM3 score and principal component 1 in the immune landscape. (**D**) Top 10 hub genes in GM3.

Identifying the functional modules of immune-related genes in STS patients is conducive to understanding the characteristics of each IS. Therefore, by applying consensus clustering, we defined six robust GMs (Figure S7). Through GO enrichment analysis, we annotated the functions: GM1—cytokine secretion, GM2—cell proliferation, GM3—T cell, GM4—chemotaxis, GM5—TNF, and GM6—mesenchyme development (Table S3). The expression patterns of ISs in TCGA cohort differed significantly across the GMs (Figure 7A,B). For example, IS2 and IS4 have the highest expression scores in GM3, while IS1 has the highest score in GM4, IS3 has the highest score in GM1, and IS6 has the highest expression score in GM6. Similar trends were also observed in the GEO cohort (Figure S8), further demonstrating the reliability of GMs. Subsequent univariate prognostic analysis suggested that GM3 could be associated with OS and RFS in patients with STS (Table S4). Through multivariate analysis with age, FNCLCC grade, gender, radiotherapy, pharmaceutical therapy, and pathological tumor size as covariates, it was further determined that GM3 score is an independent prognostic factor for STS patients (Table S5). Patients in low GM3 score group have a significantly worse prognosis than patients in a high GM3 score group (Figure 8A). Furthermore, it is noteworthy that GM3 is not only associated with T cell activation, but also closely related to leukocyte function (Figure 8B). Interestingly, GM3 was also found to be significantly negatively correlated with principal component 1 (horizontal axis) of the immune landscape (Figure 8C). Since activation and infiltration of various immune cells and immunosuppressive TIMEs both have important implications for the efficacy of mRNA vaccines, patients with a high GM3 expression score may not be suitable for mRNA vaccines. To identify hub genes in GM3 for use as mRNA biomarkers to enhance clinical applicability, the PPI network of GM3 was first constructed using the STRING tool (Figure S9), and the top 10 hub genes in GM3 were subsequently identified using the cytoHubba plugin in Cytoscape software (Figure 8D). These hub genes are potential biomarkers for identifying STS patients suitable for mRNA vaccines.

## 4. Discussion

The recent impressive efficacy of mRNA vaccines in the COVID-19 epidemic has provoked an explosion in preclinical and clinical research into infectious diseases and oncology [22]. The development of mRNA tumor vaccines relies on the identification of effective tumor antigens. Recently, Huang et al. identified potential tumor antigens for mRNA vaccine development by identifying amplified and mutated genes and overexpressed and mutated genes in cholangiocarcinoma and pancreatic adenocarcinoma, respectively [47,48]. In the present study, the above-mentioned methods were combined, and four potential tumor antigens (HLTF, ITGA10, PLCG1, and TTC3) were identified by screening key genes that were amplified, mutated, and overexpressed. These four antigens were also closely related to the infiltration of APCs and the oncogenesis and prognosis of STS. Although further preclinical evaluation is still needed, previous reports confirm their potential as targets for the development of anti-STS mRNA vaccines. For example, HLTF is involved in the RAD6-RAD18-dependent DNA damage tolerance pathway, and the alternation of HLTF expression in cancer is associated with poor prognosis [49]. In high-grade MFS, it was shown that ITGA10 promotes tumor cell survival through the activation of TRIO-RAC-RICTOR-mTOR signaling, and is thus a potential therapeutic target [50]. In addition, ITGA10 is also a prognostic marker for UPS and MFS [51]. PLCG1 is frequently mutated in angiosarcoma, increasing resistance to apoptosis and the invasiveness of endothelial cells [52,53]. Moreover, PLCG1 is also involved in the B-cell receptor signaling pathway and Fc-epsilon RI signaling pathway in osteosarcoma cells [54]. TTC3 is a ubiquitin E3 ligase that promotes ubiquitinated degradation and the phosphorylation of Akt [55], and is related to the regulation of the cancer cell proliferation rate and the tumor heterogeneity mediated by the β1-integrin/FAK/mTORC2/AKT1-related signaling pathway [56]. Notably, previous studies have shown that tumor antigens can also be generated through post-translational modifications, including, but not limited to, glycosylation, phosphorylation, and acetylation [57,58]. Due to the lack of relevant data, we could not analyze the tumor antigens generated by post-translational modifications in the present study, which deserves more in-depth study in future tumor antigen identification.

The survival benefits of patients receiving immunotherapy are usually limited to a small number of patients [59]. An enhanced understanding of the TIME and immune status of STS patients is necessary for the beneficial use of immunotherapy, including mRNA vaccines. In this study, we further identified six robust ISs based on the immune genes expression profile of STS and validated them with an independent cohort. IS1–6 have different immune statuses and TIMEs, suggesting different responses to ICIs and mRNA vaccines. Specifically, IS2 and IS4 had high enrichment scores for immunostimulatory and immunosuppressive cells, as well as high scores for leukocyte fraction, stromal fraction, and IFN-γ response, and were identified as hot but suppressive phenotypes. The prevailing view is that hot tumors are the ones most likely to benefit from ICI therapy [60]. Since IS2 and IS4 have pre-existing immune infiltration and highly expressed ICP-related genes, the use of ICI to improve immunosuppressed TIMEs and thus enhance the pre-existing antitumor immunity might be beneficial in a combinatory approach, as only a small proportion of STS patients benefited from ICI monotherapy [14,61,62]. In clinical trial SARC028, 40% of UPS and only 20% of DDLPS patients responded to ICI therapy [14]. Correspondingly, approximately 40% of UPS and 35% of DDLPS patients in our study were classified as IS2 and IS4. This demonstrates the potential guiding significance of ISs for immunotherapy strategies in STS patients. In contrast to IS2 and IS4, IS3 and IS6 were identified as cold tumors, characterized by low immune cell infiltration, low scoring for leukocyte fraction, stromal fraction, and TGF-β response, etc. Notably, in clinical trials, LMS failed to respond to nivolumab [63] and pembrolizumab treatment [14]. In TCGA and GEO cohorts, the vast majority of LMS are IS3, a cold immunophenotype with insufficient immune cell infiltration; hence, ICI treatment alone could not be successful in LMS. Our study explains, to some extent, the reason for the failure of LMS treatment in previous clinical trials. Similarly, in clinical trial SARC028, only one of the 10 SS patients responded to pembrolizumab [14], while in another study of anti-CTLA4 (ipilimumab) treatment, all six SS patients failed to respond [64]. All SS patients were classified in our investigation as IS6, another immune cold phenotype, explaining the non-responsiveness of SS to single ICI therapy. Therefore, the use of mRNA vaccines in IS3 and IS6 to induce immune cell infiltration by activating the patients’ immune systems might be a promising approach. For IS1 with a cold and suppressed TIME, determined by a low score for CD8+ T cells, leukocytes, lymphocyte infiltration, and IFN-γ response, and a high score for TGF-β response, M2 macrophages, and Th2 cells, the use of mRNA vaccines combined with ICIs might be able to reinvigorate patients’ immune system and counteract immunosuppression. IS5 exhibited moderate immune cell infiltration as well as high intra-tumor heterogeneity, TGF-β response, and Th2 cells, representing a complex and suppressive TIME. Standard but also novel approaches, such as cancer vaccines, anti-TGF-β therapy [65], chimeric antigen receptor T cell therapy [66], or oncolytic virotherapy [67], might complement ICI treatment in these sarcoma patients.

While the immune landscape recapitulated the cluster distribution of ISs in general, it also reveals the intra-cluster heterogeneity within the ISs, which has not been reflected in immunophenotyping. For example, IS2 was further divided into two subgroups, with IS2B having a colder TIME and lower immunosuppression compared to IS2A. Thus, IS2B may be a more promising candidate for mRNA vaccine attempts. Similarly, IS4B and IS5B both have a colder TIME compared to IS4A and IS5A, indicating a worse response to ICI, but also possibilities for personalized mRNA vaccines. In fact, previous reports consider STS as immunologically inert or “cold” [68].

Previous results show that IS2 and IS4 are correlated with GM3. The functional GM3 is critical for the prognosis of STS patients and is closely associated with T cells as well as leukocyte function. Based on this result, patients with high GM3 gene expression may have an immunologically hot but suppressed TIME, indicating a worse reactivity for single mRNA vaccine treatment. To enhance clinical applicability and use for preliminary screening, 10 hub genes of GM3 were identified as negative biomarkers for an mRNA vaccine response. Although the tumor antigens and ISs identified in this study require further preclinical and clinical studies for validation, they still provide references and fundamental information for the future development of mRNA cancer vaccines and the selection of STS patients for individualized immunotherapy strategies.

## 5. Conclusions

HLTF, ITGA10, PLCG1, and TTC3 are potential tumor antigens of STS for the development of mRNA vaccines. STS patients with an IS3 or IS6 are most likely to benefit from mRNA vaccination. This study provides a conceptual framework for further research into anti-STS mRNA vaccines and the selection of suitable patients for individualized immunotherapy approaches in STS.

## Data Availability

All data generated and described in this article are available from the corresponding web servers and are freely available to any scientist wishing to use them for noncommercial purposes, without breaching participant confidentiality. Further information is available from the corresponding author on reasonable request.

## Supplementary Materials

The following supporting information can be downloaded at: https://www.mdpi.com/article/10.3390/cancers14020448/s1, Figure S1: Experimental setup, Figure S2: Identification of potential overexpressed tumor antigens in STS, Figure S3: Identification of tumor antigens associated with STS prognosis, Figure S4: Identification of tumor antigens associated with infiltration of antigen-presenting cells in the GEO cohort, Figure S5: Association between immune subtypes and immunomodulators in STS, Figure S6: Molecular characteristics of immune subtypes in STS, Figure S7: Identification of potential immune-related gene modules in STS, Figure S8: Functional immune gene modules in the GEO cohort, Figure S9: The protein–protein interaction network of GM3, Table S1: Characteristics of STS samples in TCGA and GEO cohorts, Table S2: IGP was estimated for each immune subtype in the validation cohort, Table S3: Functional enrichment analysis of gene modules, Table S4: Univariate analysis of the prognostic value of gene modules in terms of OS/RFS in STS, Table S5: Multivariate analysis of the prognostic value of gene modules in terms of OS/RFS in STS.
