# Supplementary material for: Identification of Tumor Antigens and Immune Subtypes for the Development of mRNA Vaccines and Individualized Immunotherapy in Soft Tissue Sarcoma
Source: Cancers (Basel). 2022 Jan 17;14(2):448. doi: 10.3390/cancers14020448 (PMC8774220; doi:10.3390/cancers14020448)
Supplement: Supplementary file 1 [file cancers-14-00448-s001.zip › cancers-1524716-supplementary.pdf]

Supplementary materials

Identification of Tumor Antigens and Immune Subtypes for the Development of mRNA Vaccines and Individualized Immunotherapy in Soft Tissue Sarcoma

Changwu Wu, Yingjuan Duan, Siming Gong, Georg Osterhoff, Sonja Kallendrusch and Nikolas Schopow

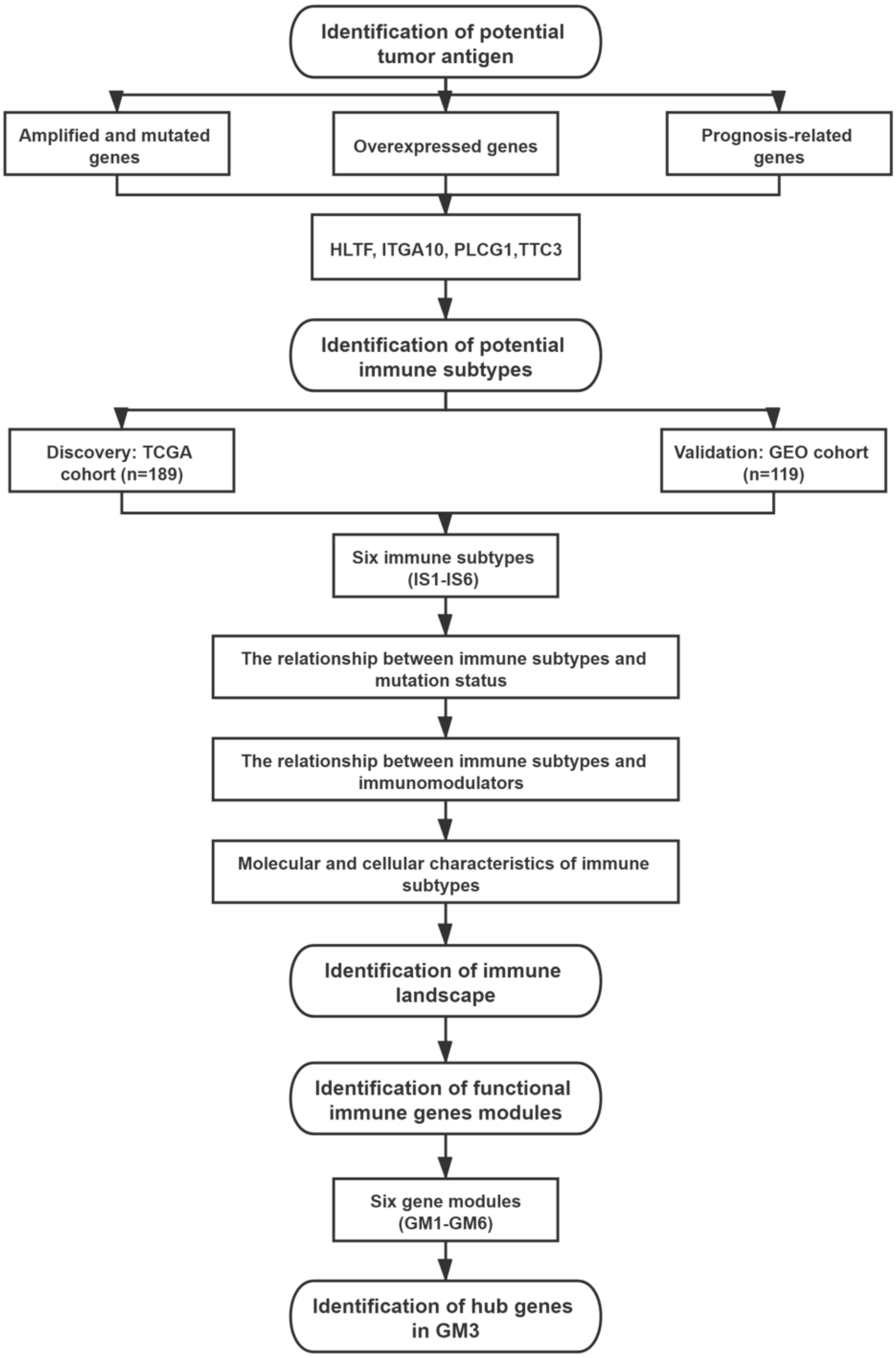

Figure S1. Experimental Setup.

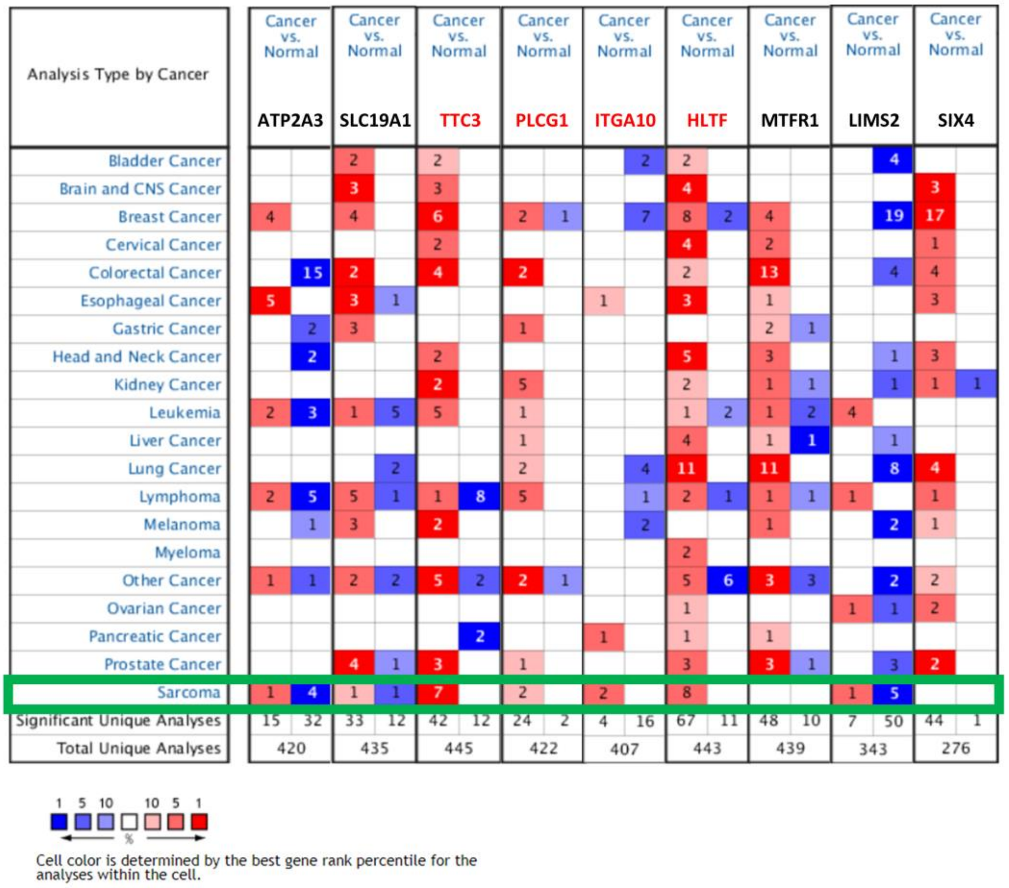

Figure S2. Identification of potential overexpressed tumor antigens in STS. Four potential tumor antigens overexpressed in STS compared to normal tissue in the Oncomine database. The red indicates that the gene expression is up-regulated and the bule indicates that the gene expression is down-regulated. The number indicates the number of analyses.

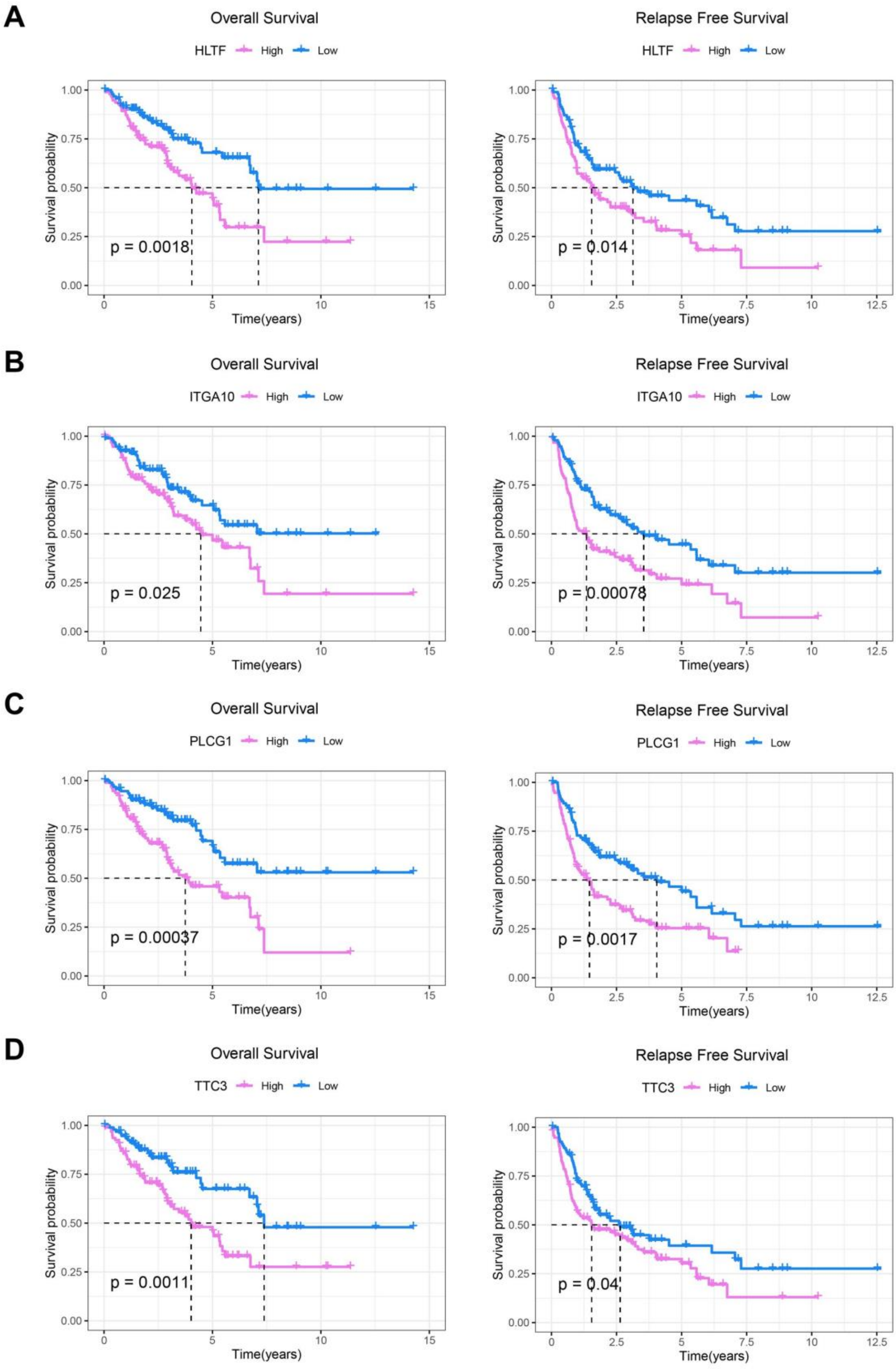

Figure S3. Identification of tumor antigens associated with STS prognosis. A-D Kaplan-Meier curves comparing OS and RFS in STS for groups with different expression of HLTF (**A**), ITGA10 (**B**), PLCG1 (**C**) and TTC3 (**D**).

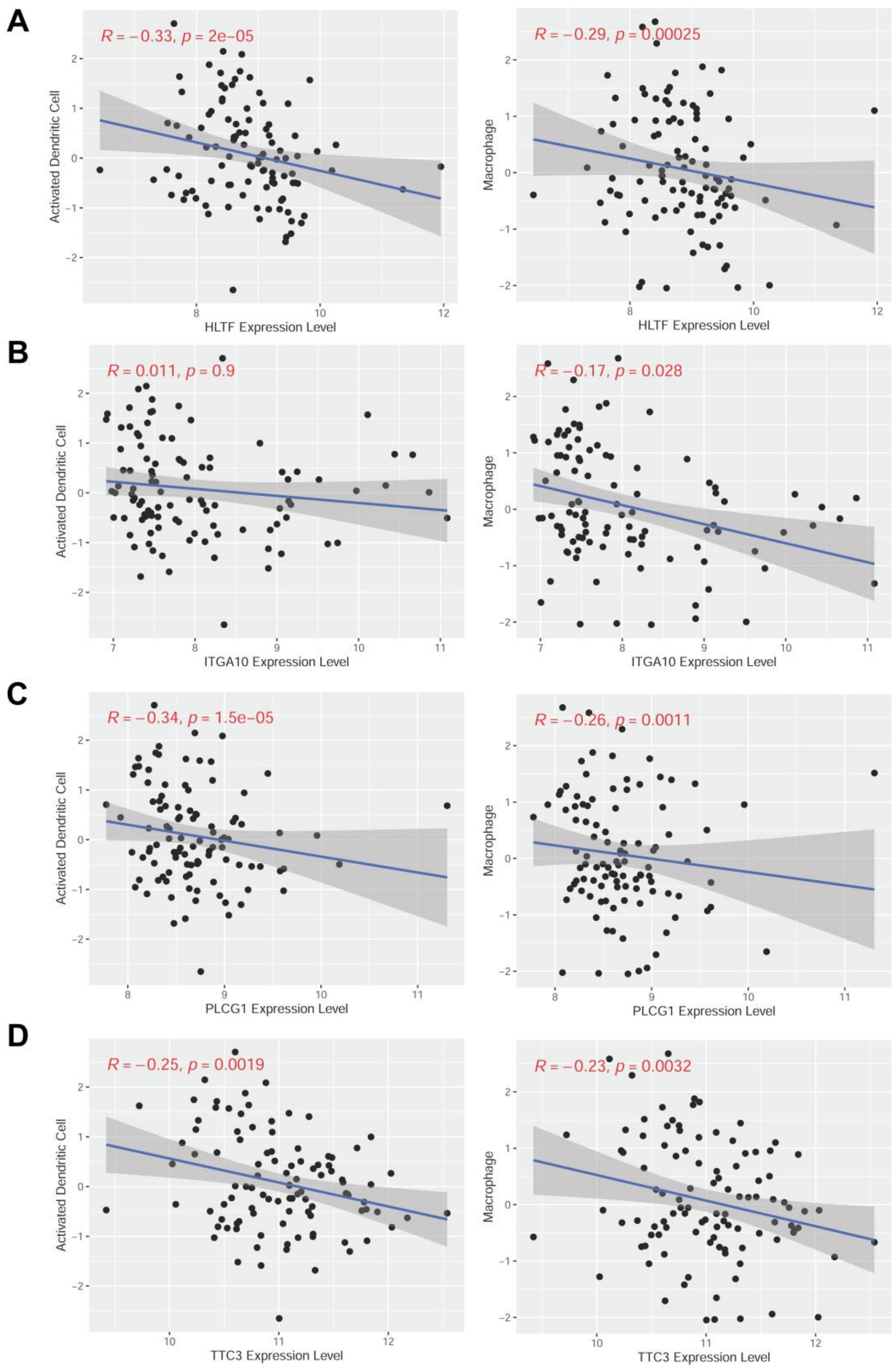

Figure S4. Identification of tumor antigens associated with infiltration of antigen-presenting cells in the GEO cohort. A-D Correlation between expression of HLTF (A), ITGA10 (B), PLCG1 (C) and TTC3 (D) and infiltration levels of dendritic cells and macrophages in STS.

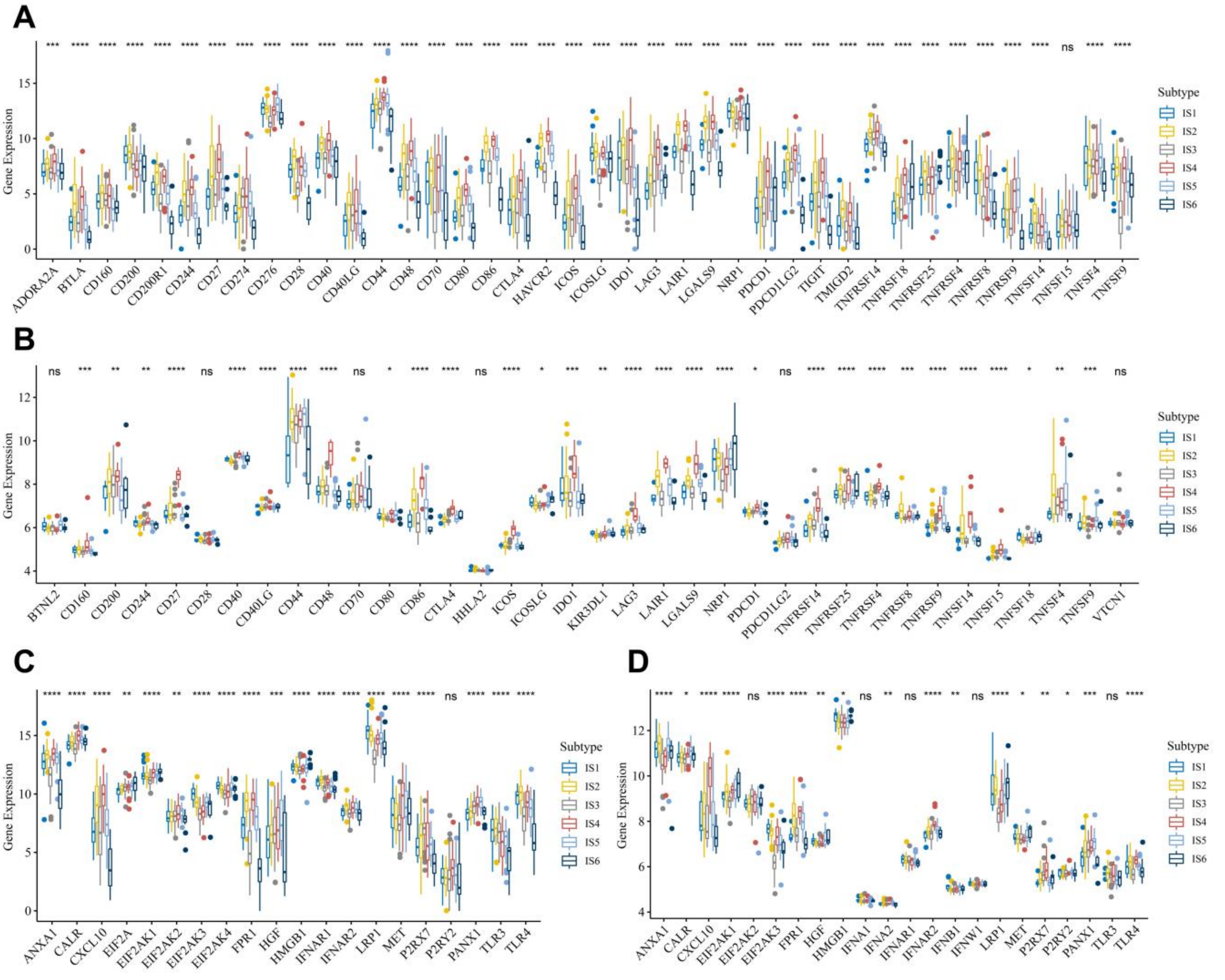

Figure S5. Association between immune subtypes and immunomodulators in STS. A,B Differences in expression levels of ICP-related genes among STS immune subtypes in TCGA (**A**) and GEO (**B**) cohorts. **C,D** Differences in expression levels of ICD-related genes among STS immune subtypes in TCGA (**C**) and GEO (**D**) cohorts. * $p < 0.05$, ** $p < 0.01$, *** $p < 0.001$, **** $p < 0.0001$.

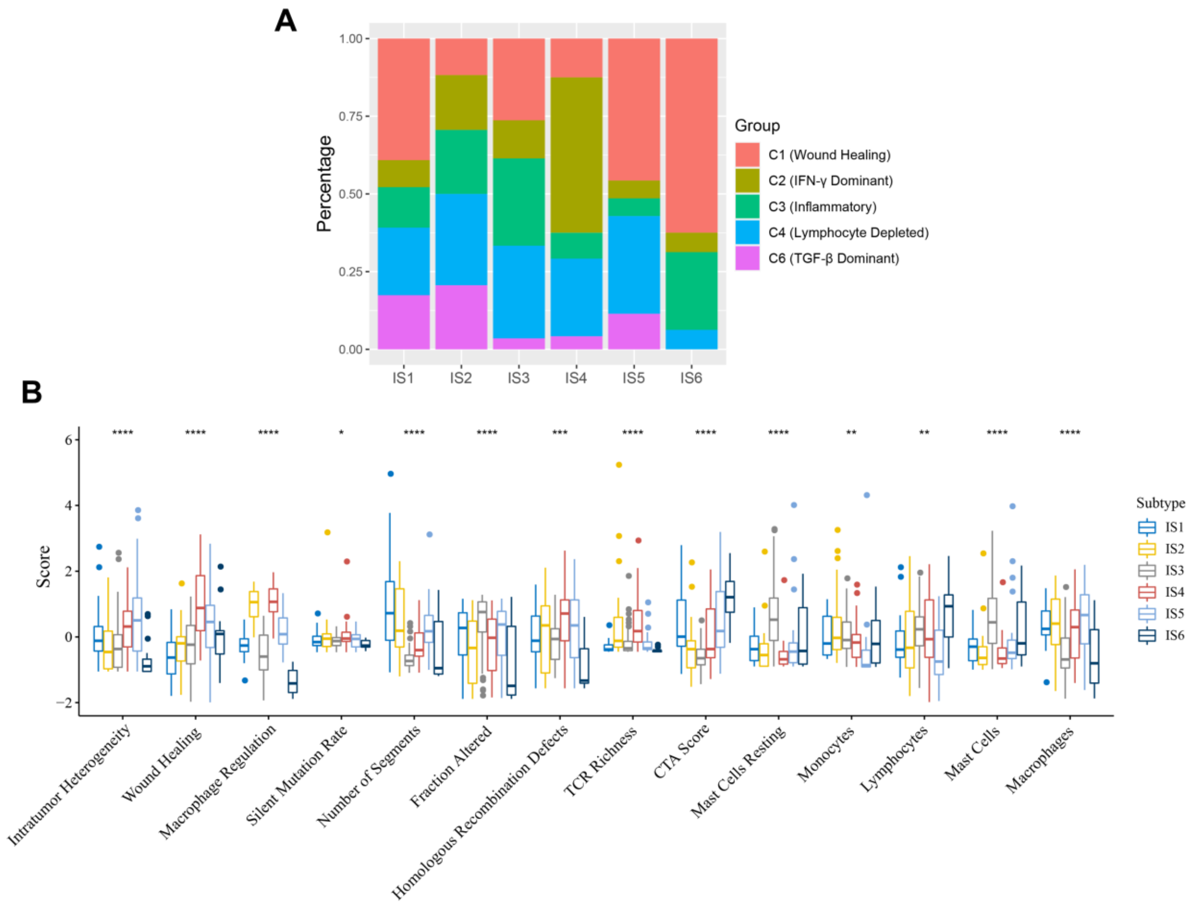

Figure S6. Molecular characteristic of immune subtypes in STS. A The distribution of six pancaner immune subtypes, defined by Thorsson et al., in the six STS immune subtypes. **B** Immune-related molecular signatures with significant differences among STS immune subtypes. * $p < 0.05$, ** $p < 0.01$, *** $p < 0.001$, **** $p < 0.0001$.

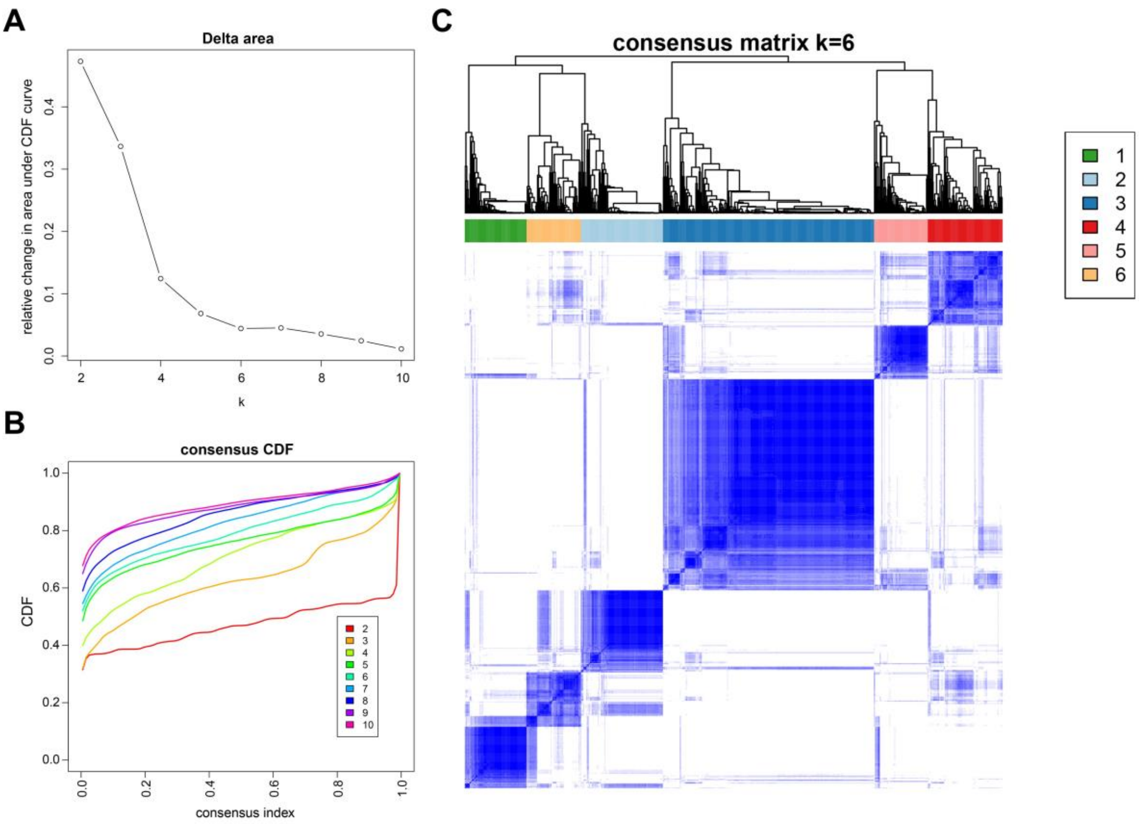

Figure S7. Identification of potential immune-related gene modules in STS. A-C Delta area curve (**A**), cumulative distribution function curve (**B**) and consensus heatmap (**C**) of immune-related gene expression profile in TCGA cohort.

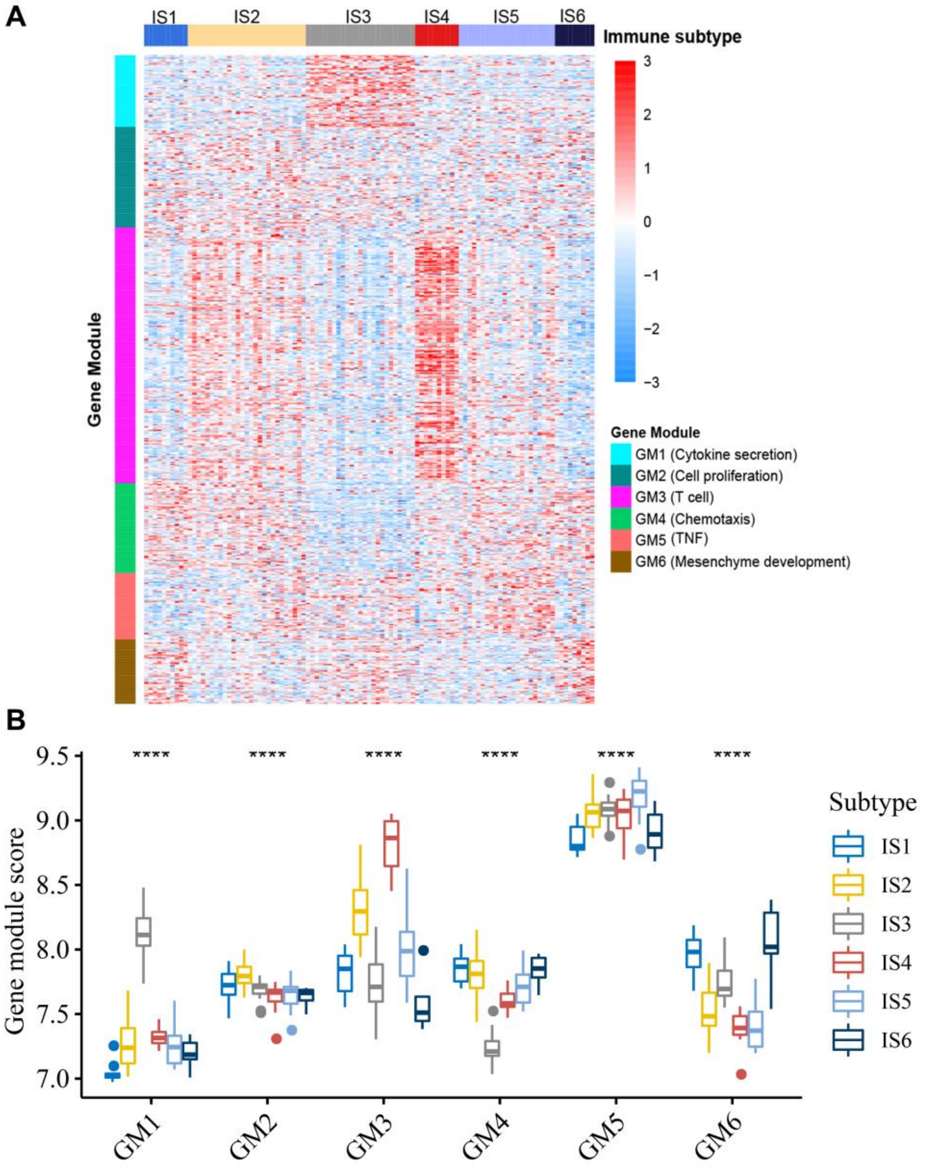

Figure S8. Functional immune genes modules in the GEO cohort. A Heatmap of ISs and GMs in the GEO cohort. Genes are ordered based on the GMs, and patients are arranged based on their ISs. **b** Box plots of the GM expression patterns of six immune subtypes in the GEO cohort. **** $p < 0.0001$.

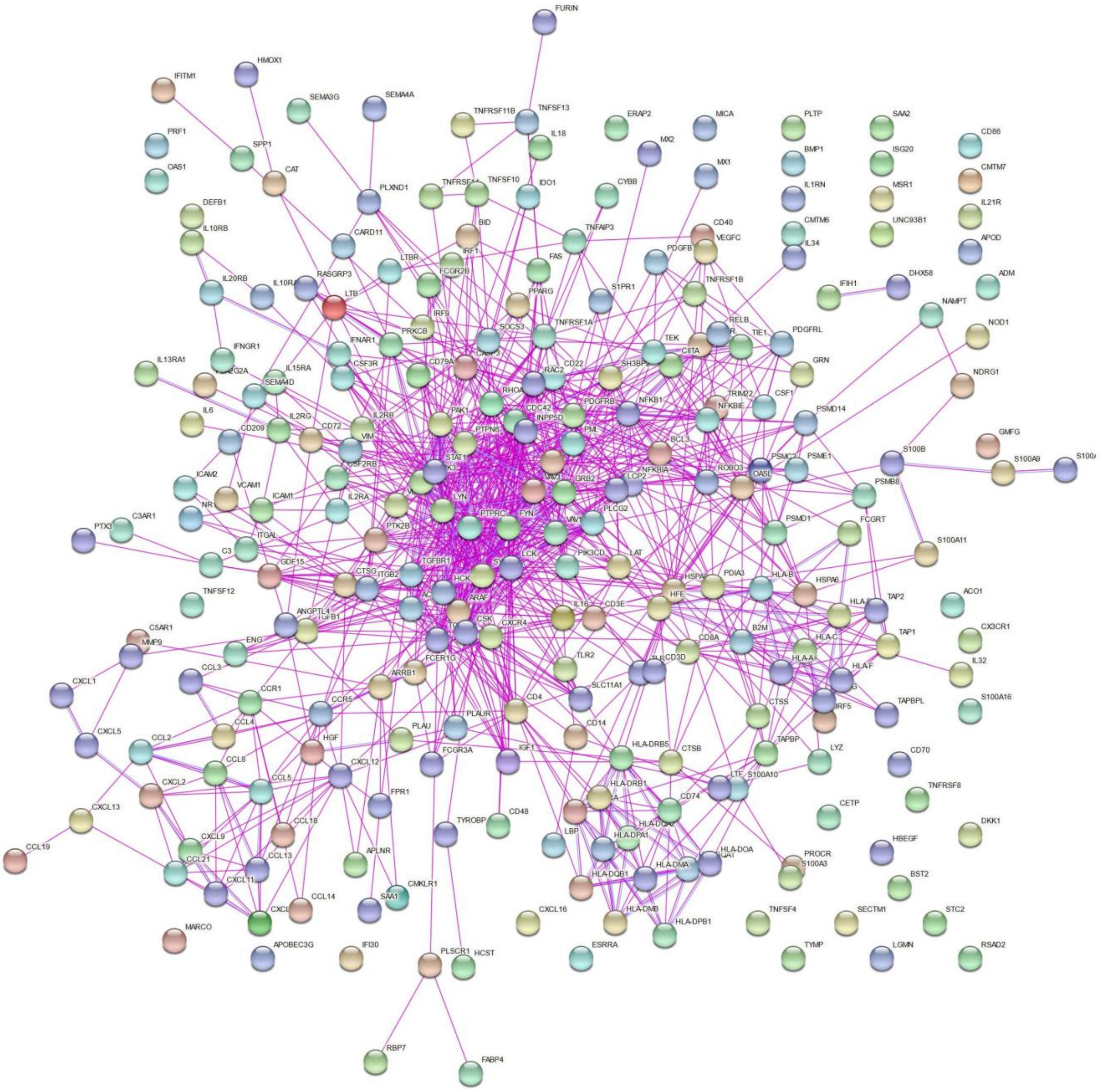

Figure S9. The protein-protein interaction network of GM3. Each node represents each protein and the line represents the interaction between the proteins.

Table S1. Characteristics of STS samples in TCGA and GEO cohorts.

Characteristics	TCGA (n=189)	GEO (n=103)
**Histological type**		
DDLPS	49 (25.93 %)	46 (44.66 %)
LMS	68 (35.98 %)	26 (25.24 %)
UPS	41 (21.69 %)	NA
MFS	17 (8.99 %)	31 (30.10%)
SS	10 (5.29 %)	NA
MPNST	4 (2.12 %)	NA
**Pathologic tumor size**		
≤ 10.5 cm	95 (50.26 %)	NA
> 10.5 cm	94 (49.74 %)	NA
**Radiotherapy**		
Yes	54 (28.57 %)	NA
No	135 (71.43 %)	NA
**Pharmaceutical therapy**		
Yes	45 (23.81%)	NA
No	144 (76.19%)	NA
**FNCLCC grade**		
1	10(5.29%)	NA
2	105 (55.56%)	NA
3	74 (39.15%)	NA
**Vital status**		-
Alive	117 (61.90%)	NA
Dead	72 (38.10%)	NA
**Relapse status**		
Relapse	114 (60.32%)	NA
Non- Relapse	75 (39.68%)	NA

DDLPS: dedifferentiated liposarcoma; LMS: leiomyosarcoma; UPS: undifferentiated pleomorphic sarcoma; MFS: myxofibrosarcoma; SS: synovial sarcoma; MPNST: malignant peripheral nerve sheath tumor; NA: no data or data unavailable.

Table S2. IGP was estimated for each immune subtype in the validation cohort.

Immune subtype	IS1	IS2	IS3	IS4	IS5	IS6
IGP value	0.857	0.625	1.000	0.640	0.588	0.667

Table S3. Functional enrichment analysis of gene modules.

Gene module	Annotation	Top 5 enriched biological processes
GM1	Cytokine secretion	GO:0009755~hormone-mediated signaling pathway<br>GO:0007162~negative regulation of cell adhesion<br>GO:0030522~intracellular receptor signaling pathway<br>GO:0043401~steroid hormone mediated signaling pathway<br>GO:0050707 ~regulation of cytokine secretion
GM2	Cell proliferation	GO:0050673~epithelial cell proliferation<br>GO:0033002~muscle cell proliferation<br>GO:0048638~regulation of developmental growth<br>GO:0048015~phosphatidylinositol-mediated signaling<br>GO:0048017~inositol lipid-mediated signaling
GM3	T cell	GO:0050900~leukocyte migration<br>GO:0042110~T cell activation<br>GO:0060326~cell chemotaxis<br>GO:0030595~leukocyte chemotaxis<br>GO:0034341~response to interferon-gamma
GM4	Chemotaxis	GO:0050920~regulation of chemotaxis<br>GO:0061138~morphogenesis of a branching epithelium<br>GO:0001763~urogenital system development<br>GO:0001763~morphogenesis of a branching structure<br>GO:0048608~reproductive structure development
GM5	TNF	GO:0033209~tumor necrosis factor-mediated signaling pathway<br>GO:0002479~antigen processing and presentation of exogenous peptide antigen via MHC class I, TAP-dependent<br>GO:0042590~antigen processing and presentation of exogenous peptide antigen via MHC class I<br>GO:0002833~positive regulation of response to biotic stimulus<br>GO:0002223~stimulatory C-type lectin receptor signaling pathway

GM6	Mesenchyme development	GO:0060485~mesenchyme development<br>GO:0048762~mesenchymal cell differentiation<br>GO:0050919~negative chemotaxis<br>GO:0048843~semaphorin-plexin signaling pathway<br>GO:0048863~stem cell differentiation

Table S4. Univariate analysis of the prognostic value of gene modules in terms of OS/RFS in STS.

Gene Module (GM)	Overall survival			Relapse-free survival		
	**HR**	**95%CI**	***P*-value**	**HR**	**95%CI**	***P*-value**
GM1 score	0.80	0.63~0.99	0.049	0.92	0.77~1.10	0.368
GM2 score	0.89	0.54~1.46	0.646	0.93	0.62~1.40	0.716
GM3 score	0.79	0.65~0.97	0.027	0.859	0.73~1.01	0.078
GM4 score	1.23	0.91~1.66	0.175	1.01	0.79~1.27	0.966
GM5 score	0.89	0.53~1.53	0.690	0.993	0.65~1.52	0.972
GM6 score	1.22	0.91~1.65	0.184	1.05	0.83~1.32	0.689

Table S5. Multivariate analysis of the prognostic value of gene modules in terms of OS/RFS in STS.

Characteristics	Overall survival			Relapse-free survival		
	**HR**	**95%CI**	***P*-value**	**HR**	**95%CI**	***P*-value**
Age (Continuous)	1.03	1.01~1.05	0.007	1.01	0.99~1.00	0.390
Gender (Male vs. Female)	0.93	0.55~1.59	0.794	1.24	0.83~1.85	0.301
Pathological tumor size (cm)	1.05	1.04~1.08	<0.001	1.04	1.02~1.14	<0.001
Radiotherapy (Yes vs. No)	0.70	0.36~1.34	0.282	1.04	0.67~1.72	0.894
Pharmaceutical therapy (Yes vs. No)	1.73	0.95~3.13	0.072	1.92	1.21~3.01	0.005
FNCLCC grade (3 vs. 1/2)	1.43	0.79~2.61	0.240	1.592	1.56~2.50	0.064
GM1 score	0.79	0.49~1.25	0.306	0.96	0.67~1.40	0.802
GM2 score	1.60	0.56~4.55	0.379	1.48	0.68~3.21	0.319
GM3 score	0.60	0.41~0.87	0.008	0.70	0.55~1.00	0.053
GM4 score	1.17	0.61~2.22	0.641	0.99	0.59~1.71	0.970
GM5 score	1.70	0.53~5.39	0.369	1.71	0.69~4.32	0.249
GM6 score	0.94	0.57~1.56	0.822	0.93	0.62~1.44	0.727
